# Safe Harbor: Protecting Ports with Shipboard Fuel Cells

**DOI:** 10.1289/ehp.114-a236

**Published:** 2006-04

**Authors:** David A. Taylor

In September 2005, the city of Los Angeles announced a mandate to cut the air pollution from its harbor. The head of the city’s harbor commission, S. David Freeman, gave the port managers a sobering directive: “Start acting like our lives are depending on it,” he told them, according to the 29 September 2005 *Los Angeles Times*, “because our lives do depend on it.” Three months later, the California Air Resources Board adopted rules that require ships within 24 miles of the state’s coast to reduce diesel emissions to 2001 levels within the next four years. These developments have shippers scrambling to find a way to cut emissions. One of the main technologies attracting their attention is the use of fuel cells.

## The Full Import

Each year, the Port of Los Angeles—occupying 7,500 acres and 43 miles of waterfront—handles more than 162 million metric revenue tons of cargo (measured as 1,000 kilograms or 1 cubic meter, whichever is larger). With the increase in Pacific commerce, port diesel emissions have increased 60% since 2001, and the port complex has in 10 years become the single largest air polluter in the Los Angeles basin, according to the 25 September 2005 edition of the *Los Angeles Times*.

Nearby residents blame the port for illnesses ranging from asthma to cancer, according to Diane Bailey, head of the Health and Environment Program for the Natural Resources Defense Council (NRDC) in San Francisco. Studies have linked particulate matter from diesel fumes to respiratory illness and cancer.

In an August 2004 report titled *Harboring Pollution: Strategies to Clean Up U.S. Ports*, the NRDC noted that besides direct threats to human health, growing harbor traffic could increase regional smog, threaten water quality and public lands, and increase noise and light pollution. With three of the country’s five largest harbors in California, Bailey says, cutting port pollution and the health impacts on surrounding communities is a “huge priority” for the state.

The source of most ship-related emissions is Bunker C fuel, a diesel that produces a thick, sticky residue. (Bunker C gets its name from the era when steamships were fired by coal stored in bunkers. When ships shifted to diesel, crews still used the term “bunker” to include liquid fuel tanks.) Ships in port can also use a kind of extension cord plugged in to land power supplies, but Scott Samuelsen, director of the National Fuel Cell Research Center at the University of California, Irvine, says these cords can be somewhat dangerous if they get tangled during loading or unloading. Therefore, most ships in port use diesel engines to provide “hoteling” power—basic lighting, heating, ventilation, and light electricity.

## The Beauty of Fuel Cells

Fuel cells are cleaner than diesel turbines and other internal combustion engines. Samuelsen explains that fuel cells convert energy from hydrogen directly to electricity without combustion; the only residues are water and heat.

A fuel cell works using the same electrochemical reaction as the battery under a car hood, Samuelsen explains. But whereas the battery in your car primarily stores energy while the engine is turned off, a fuel cell reaction provides energy continuously as hydrogen fuel encounters oxygen. Compared to internal combustion, says Samuelsen, “the fuel cell is more of a one-stop shop, one reaction.” And with fewer moving parts, there’s the prospect of reduced maintenance.

The NRDC recommended fuel cells as a quieter, cleaner, more efficient power source for ships in *Harboring Pollution*. Since the report appeared, Bailey says other technologies have gained momentum, at least in the short run. Among them are diesel–electric hybrids, more efficient versions of the locomotive engine, which can cut emissions by 90% compared with the old diesel engines used to assemble freights in port rail yards. Another alternative is the gas turbine engine, which has strong marketplace advocates.

In Los Angeles, though, says Samuelsen, “the fuel cell will probably be the preferred choice.” He adds that California’s regulatory action will likely point the direction for maritime energy use elsewhere. As in the drive toward greater fuel efficiency in automobiles, Samuelsen suggests, the state’s regulatory process will likely drive national technological advances.

## Contemplating Naval Studies

The U.S. Navy has been exploring shipboard use of fuel cells for some time, according to Anthony Nickens, a program officer with the Office of Naval Research (ONR) in Arlington, Virginia. Nickens says the Navy is very interested in the technology for its efficiency and low emissions. “There’s no [nitrogen oxide emissions] coming out, there’s no flame,” he says. Fuel cells also permit a “distributed” power system design; they can be located at different points in the ship, away from the ship’s principal exhaust stack system, unlike conventional power-generation and propulsion engines. This flexibility can improve ship survivability in the event of an accident or enemy attack, according to an ONR press release from February 2004.

So far, fuel cells are still in a demonstration phase supervised by ONR’s science and technology staff. “I think we can get there in five, seven years,” says Nickens of the Navy’s plans for fuel cell–powered vessels. One demonstration for the Navy by Sandia National Laboratories and Plug Power, a company based in New York, was completed in August 2005. The project tested 20 fuel cells at naval support sites in California, New York, and Hawaii, and proved their ability to provide heat and power for land-based functions. Abbas Ali Akhil, Sandia’s energy analyst on the project, says a final report on that demonstration will be completed later this spring. Meanwhile, test results from locations in three states are available at the Sandia website [see Suggested Reading below].

Steven Eschbach, director of investor relations and communications for FuelCell Energy, based in Danbury, Connecticut, says his company, too, has been working for several years to provide the Navy with a land-based demonstration of shipboard fuel cells for providing hoteling power to stealth destroyers. Eschbach says the silence of fuel cells is an important benefit for stealth destroyers, but it also helps that these power sources are more efficient (47% electrical efficiency—the portion of total energy in the reaction that is translated into usable electrical power—compared to 30–40% for diesel engines) and 99% cleaner, especially in terms of nitrogen oxide, sulfur oxide, and particulate emissions. The company is currently testing peripheral components of the fuel cell power plant and will soon integrate those with a fuel cell stack for complete system verification.

Another key advantage to the fuel cell approach is that the hydrogen needed for the electrochemical power generation is internally purified in the fuel cell module. “We don’t need a hydrogen infrastructure for our fuel cells to operate,” Eschbach says. The company plans to continue testing this spring, followed by delivery of the fuel cell system later this year pending funding from the Navy.

The German Navy also is exploring fuel cells and in October 2005 commissioned two new fuel cell–powered submarines from Siemens KWU, according to a 23 January 2006 report by the online industry publication *Fuel Cell Today*. The Siemens model uses a solid polymer electrolyte membrane (PEM) fuel cell to direct hydrogen ions to a cathode for reaction with oxygen inside a pressurized housing. PEM cells, operating at temperatures less than 80ºC, are reportedly 60% efficient, according to *Fuel Cell Today*.

## Commercial Applications

For commercial ships, Samuelsen expects to see design prototypes in about five years, spearheaded by one or two shipping companies that pave the way. He also expects the shift to be gradual, with “a few decades before the momentum grows,” he says. After all, the new laws dictate reduced ship emissions, but they don’t dictate fuel cells as the only way to get there.

For cruise operators wanting to spotlight an environment-friendly fleet and give passengers a quieter cruise experience, fuel cells may be especially appealing. Calá Corporation, a cruise line operator based in Titusville, Florida, has plans to build three cruise ships equipped with fuel cells, says CEO Joseph Calá. The fuel cells will provide hoteling power for electricity as well as power at low speeds (under 8 knots). Calá expects the first vessel to be ready by 2008. Each will be equipped with about 20 cells providing 500 kilowatts apiece. Calá estimates that the cells will save perhaps $1 million in fuel each year. Cruising at 16 knots, he says a ship can burn up to $35,000 in fuel a day.

Calá, who became interested in fuel cells’ promise 12 years ago, maintains that nautical engineers aren’t moving fast enough in furthering the technology. “They need to bring in people with vision,” he says. “They need to expand their minds and imagination.”

Several technical challenges remain, notes John Weidner, a chemical engineering professor at the University of South Carolina. Unlike other forms of energy, fuel cells don’t offer economies of scale for making the large units needed for ships. This is due to the simplicity of the fuel cell chemical reaction. There are no moving parts you can accelerate for a bigger bang; the energy output is strictly related to the size of the cell. “You can make them big, but if you make it ten times bigger, it costs ten times as much,” says Weidner. Furthermore, in some applications, durability of the fuel cell’s electrolyte is still an issue. For some types of fuel cells, including molten carbonate cells, the high temperatures involved decrease cell life.

Despite these challenges, the technology is promising for maritime use. As more research comes online, we can likely expect to see fuel cells surging full steam ahead.

## Figures and Tables

**Figure:**
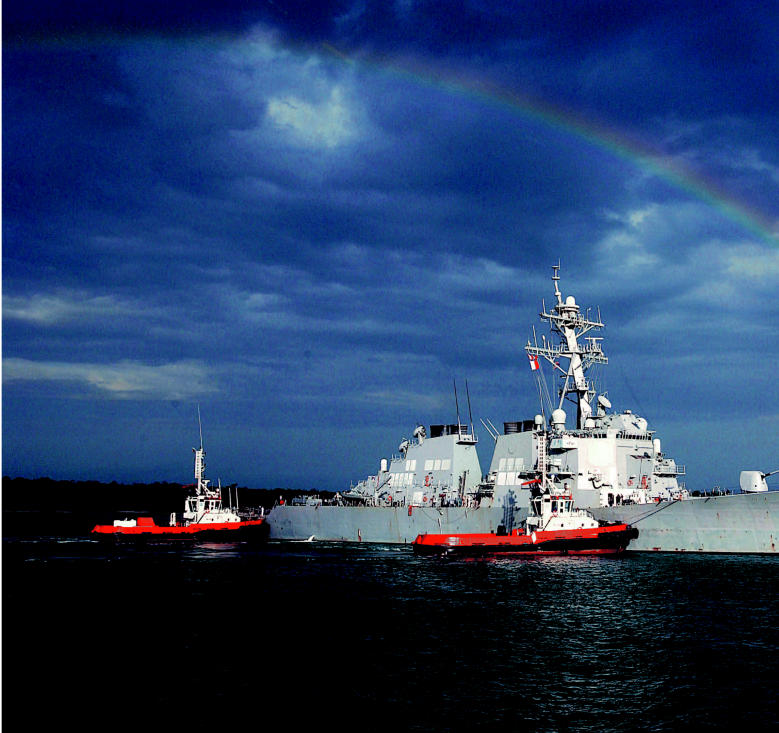
Cleaner cruising. A rainbow arches over the guided missile destroyer USS *Gonzalez* (DDG 66) as it pulls into the Port of Mombasa, Kenya. The U.S. Navy is one of several groups investigating the use of fuel cells in its fleet. Fuel cells offer the advantages of being less polluting and quieter than diesel engines.

**Figure:**
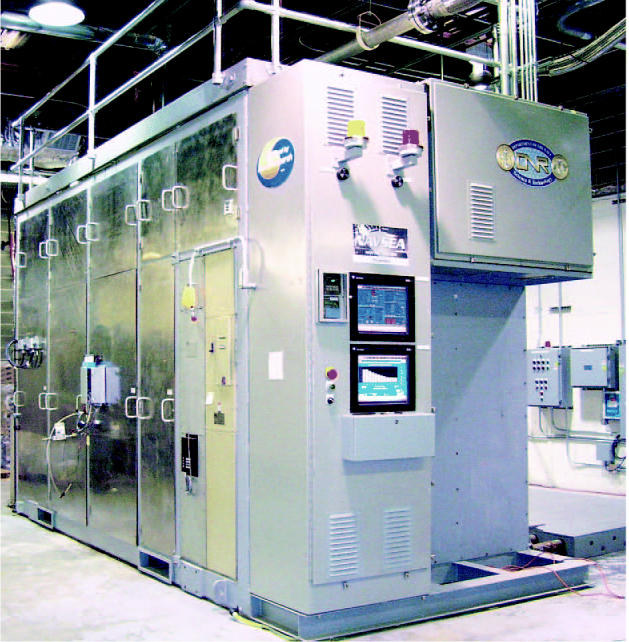
Hot box. The 625 Molten Carbonate Fuel Cell Reformer System (fuel cell stack is out of view) produces methane from high-sulfur logistics fuel to power the fuel cell stack with an expected efficiency of 47–50%. The system is being land-tested by the Office of Naval Research, and will be installed at the Naval Sea System Command’s engineering facilities in Philadelphia in 2007 for extended testing.

**Figure:**
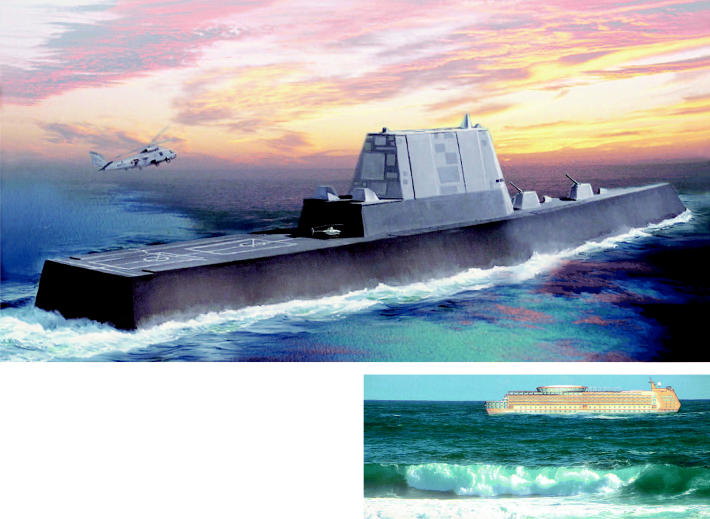
Picturing the future fleet. Artist’s concepts of two potential ships that may use fuel cells in the not-too-distant future: (above) a design by a Northrop Grumman Corporation–led team of the U.S. Navy’s 21st century surface combatant, and (right) Calá Corporation’s fuel cell–powered luxury cruise ship.
